# Considerations for Using a Geographic Information System to Assess Environmental Supports for Physical Activity

**Published:** 2004-09-15

**Authors:** Dwayne E Porter, Karen A Kirtland, Joel E Williams, Matthew J Neet, Barbara E Ainsworth

**Affiliations:** Department of Environmental Health Sciences, Arnold School of Public Health, University of South Carolina; Prevention Research Center, Arnold School of Public Health, University of South Carolina (USC), Columbia, SC; Prevention Research Center, Arnold School of Public Health, University of South Carolina (USC), Columbia, SC; Belle W. Baruch Institute for Marine and Coastal Sciences, USC, Columbia, SC; Department of Exercise and Nutritional Sciences, College of Professional Studies and Fine Arts, San Diego State University, San Diego, Calif

## Abstract

The use of a geographic information system (GIS) to study environmental supports for physical activity raises several issues, including acquisition and development, quality, and analysis.

We recommend to public health professionals interested in using GIS that they investigate available data, plan for data development where none exists, ensure the availability of trained personnel and sufficient time, and consider issues such as data quality, analyses, and confidentiality.

This article shares information about data-related issues that we encountered when using GIS to validate responses to a questionnaire about environmental supports for physical activity.

## Introduction

Beginning with John Snow's 19^th^-century use of maps to track the source of a cholera epidemic in London, maps have been an instrumental tool in addressing public health concerns (1). A geographic information system (GIS) is a tool that facilitates the development of dynamic maps with data integration and analysis techniques focused on public health issues such as environmental supports for physical activity (PA).

Environmental supports for PA have been well documented in the public health community (2). PA levels have been positively associated with the presence of environmental features, including sidewalks (3) and recreation facilities (4,5). Most studies that have identified associations of the environment to PA have used self-report data (2). Few PA studies have obtained objective measures of the environment using GIS (6,7). For example, GIS was used to assess elevation measures to compare terrain with trail use (6). Rather than relying on self-report measures of the environment, researchers using GIS can compare PA behavior to the actual environment (7).

GIS can be used to manipulate, analyze, and present information linked to a geographic location (8). One intriguing aspect of this technology is the limited knowledge of its capabilities and limitations in the public health field (9,10). We recently used GIS to validate responses to a questionnaire about environmental features (e.g., sidewalks, streetlights) believed to be related to PA (7). (The complete survey is available from: URL: http://prevention.sph.sc.edu/tools/docs/Env_Supports_for_PA.pdf.) The intent of this essay is to share information about data-related issues we encountered, including data acquisition and development, data quality, and GIS-based data analysis.

### Data acquisition and development

Challenges of acquiring and developing GIS data include knowledge of resources for obtaining data; agreement issues between data owner and user; knowledge of methods to develop data such as using a global positioning system (GPS) or geocoding; creation of attributes that describe the data; and trained personnel and sufficient time to conduct these activities.

Acquiring data for GIS can range from downloading Internet files to contacting government offices or companies for use of their data, which can be time consuming. In our study, we, with no formal written agreement, acquired data on roads, waterways, and public facilities from state agencies. However, we entered into written agreements with the local police departments to obtain locations of crime incidents. When we collected data on streetlights, we found that one utility company maintained data for locations of county-owned streetlights and required no written agreement and another utility company maintained data for locations of city-owned lights and required a written agreement. The written agreement was intended to ensure that the data were not used in a manner unacceptable to the data provider. Furthermore, one company was local and had data available only on paper maps, while the other company was located in a different state and maintained digital data.

When data did not exist, we used GPS to map environmental features such as trails and sidewalks. Although we preferred personnel to have preexisting knowledge of how to use GPS, we had to train some personnel, which was time consuming. When data existed only in a hard-copy form (e.g., paper maps) and GPS was not a reasonable alternative, information available from other sources was manually converted (e.g., scanning images) into a digital form for use in GIS. For example, county-maintained streetlights were manually digitized into GIS because only paper maps were available and it was not practical to apply GPS to more than 15,000 streetlights.

Another important technique in determining locations of environmental supports for PA and residential locations of survey respondents was the method of geocoding. Geocoding maps an address to geographic coordinates using a georeferenced street database (11). In our study, we geocoded addresses of crime incidents, unattended dogs, places of worship, schools, and respondents. Time constraints and knowledge of geocoding techniques are factors to consider when planning a GIS project. For example, we geocoded 1112 residential addresses but more than 20,000 crime addresses.

Once data on location of environmental supports for PA and respondents were collected and integrated into GIS, we obtained attributes about those features. Attributes are characteristics about the environment or individuals that are linked to a spatial feature (e.g., location of facility, respondent). Some of the environmental characteristics of our study were traffic volume, condition of sidewalks and recreation facilities, and opportunities for PA in schools and places of worship. We acquired annual average daily traffic counts from the South Carolina Department of Transportation (SC DOT) and conducted in-person audits to collect data related to sidewalk maintenance and public recreation facility conditions. We contacted schools and places of worship to determine if opportunities for PA were available to the public. We then linked attributes to their features (e.g., locations of roads, sidewalks, recreation facilities, schools, places of worship). Finally, survey responses were linked to the residential location of each survey respondent. In all data-collection activities, trained personnel and time availability were key elements in successfully obtaining or developing data.

### Data quality

**Figure 1:**
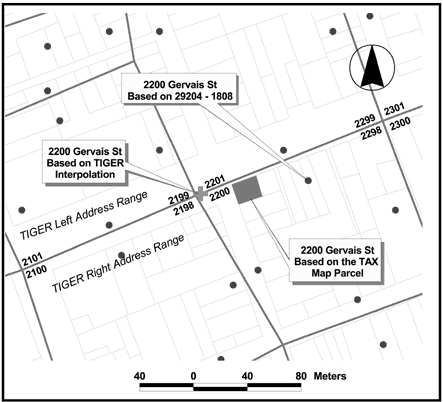
Geocoded locations of a single address using three different road files, illustrating a potential source of error in geographic information systems (GIS) (13).

The expression "garbage in, garbage out" is true of GIS: if data put into the system are inaccurate or incomplete, the GIS product will be of minimal value. GIS data quality concerns include spatial scale and spatial errors (10), incomplete data (10), temporal issues (10), and incomplete or erroneous attributes (12). In the best-case scenario, metadata should be available for all data to provide users enough information to determine data quality. In our study, the utility company that was located in a different state provided the city streetlight data, which was saved in their local coordinate system. We then reprojected the data to match the coordinate system of the study area. Road files used for geocoding addresses can also be potential sources of inaccuracy (10). Figure 1 shows a variation of approximately 20 meters between locations of an address mapped using three different road files (13). Also, road files are limited to road names and numbers; therefore, addresses without that type of information (e.g., rural routes) cannot be accurately geocoded. When an address cannot be mapped, the user needs to determine if the problem results from an incomplete road file or an inaccurate address (10,14). Temporal components of data should also be considered (10). A TIGER (Topologically Integrated Geographic Encoding and Referencing) road file, available through the 1990 U.S. Census Bureau survey, may be an inaccurate representation of current roads, because roads may have changed or new roads may have been created. Inaccuracies may be found with other nonstatic types of data, such as digital elevation models and aerial photographs.

Finally, data represented by spatial coordinates on a map in GIS also have additional information stored in an attribute table. Attributes can represent crucial information that is required in data analyses. In our study, the crime database contained features that were mapped to show the location of where a crime had occurred, but the attribute table contained characteristics about that feature, including type of crime and when the crime occurred. Because attributes provide important information about features, we checked the attribute data to ensure accuracy and completeness. For example, SC DOT traffic counts, which were attached as attributes to the road file, represented only state-maintained roads. County-maintained roads did not have traffic counts. Crime data entries were encoded and included administrative calls. Thus, we had to decode the data to reveal only crime incidents.

### Data analyses

GIS-based data analyses in our study included creating neighborhood buffers and community road networks, interpolating traffic counts, and querying and exporting attributes to determine distributions of crime data. One goal of our study was to compare perceptions of the environment to the actual environment at the neighborhood and community levels. To make these comparisons, we quantified neighborhood and community environments using GIS-based spatial analyses and network analyses (15,16). Researchers describing geographic environments measured with GIS-based tools should explain the differences between distance defined "as the crow flies" using spatial analyses and distance defined by a road network using network analyses. In the PA study, we used a half-mile buffer encircling the survey respondent's address to represent the respondent's neighborhood. In contrast, we used a 10-mile buffer encircling the respondent's address, with the buffer defined and shaped by the surrounding road network, to represent the respondent's community.

**Figure 2:**
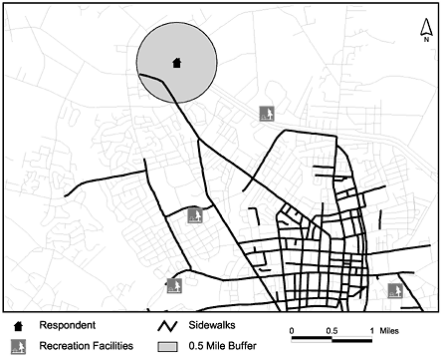
Using a half-mile buffer to represent a neighborhood around a survey respondent's home address, GIS can be used to identify a sidewalk or recreation facility in a survey respondent's neighborhood.

As part of our process of comparing perceptions of the environment to the actual environment, we integrated survey responses into GIS. Figure 2 provides an example of a respondent's neighborhood and proximity of recreation facilities and sidewalks. We compared this actual environment to the survey respondent's perception of it. These repetitive comparisons can be made manually, but it may take months. A computer program generated in GIS-supporting languages (e.g., Avenue, Visual Basic) may produce results within a short period of time, saving time and money.

**Figure 2. **Using a half-mile buffer to represent a neighborhood around a survey respondent's home address, GIS can be used to identify a sidewalk or recreation facility in a survey respondent's neighborhood.

We made some comparisons based on the presence or absence of an environmental feature, but we made others based on features that required a scale of measurement, such as heavy or light traffic based on traffic counts or safe or unsafe neighborhoods based on crime data. We used interpolation techniques to estimate traffic counts along county-maintained roads, which were not counted by SC DOT. Although interpolation techniques have been traditionally used in spatial-based analyses (17), they are not so familiar to researchers interpolating spatial data. Thus, trained and experienced personnel should be considered when using interpolation techniques.

To designate environments as safe or unsafe, we investigated geographic distributions of various types of crimes. Using codes created by the Federal Bureau of Investigation (18), crime incidents were identified by degree of violence. We examined distributions of violent and nonviolent crime incidents to classify neighborhoods as safe or unsafe. Other measures of the social environment, such as questions about trustworthy neighbors or pleasant neighborhoods, were difficult to assess using GIS. However, we were able to use mean survey responses to social questions to designate pleasant neighborhoods using GIS.

Researchers also need to ensure that the development and use of GIS data are appropriate for their research question. In our study, we developed GIS data to validate survey responses about the presence or absence of environmental supports for PA. However, researchers may also be interested in analyzing associations of GIS measures to PA. In this case, GIS measures may need to go beyond the presence or absence of features and take into account quantitative measures such as miles of trails and sidewalks or number of recreation facilities.

Although GIS is a powerful tool for assessing individual and environmental features and characteristics, there are limitations in using this technology, especially for public health studies. Available data that can be used in GIS may be incomplete or inaccurate, and sometimes data are not available. Other types of limitations include the human and monetary resources required to incorporate GIS into a public health study. For example, we initially believed that validating survey responses in the PA study using GIS would be straightforward and simple. Ultimately, five additional personnel were hired to assist the research team. A university lawyer was involved to ensure confidentiality of shared data. It took years to collect and interpret the GIS data, instead of the initially projected one year. In addition, the costs to complete the study were nearly double costs originally budgeted. Thus, insufficient knowledge of required time, personnel, or money will limit the addition of GIS into a public health study.

In our study, we had to consider data-related issues involving acquisition, development, quality, and analysis. We also had to consider issues of confidentiality and agreements with data providers. The Table summarizes key points researchers should consider when using GIS. By integrating many different types of data into GIS, we validated survey responses about environmental supports for PA. As long as users understand the capabilities and limitations of both GIS and spatial data, GIS can be a valuable tool to support improved community-level assessment and understanding of the relationships between PA and the environment.

## Figures and Tables

**Table:** Key Points to Consider in Using a Geographic Information System (GIS) to Assess Environmental Supports for Physical Activity

Ensure enough time, money, and properly trained personnel are budgeted.
Hire trained personnel who can create or collect and evaluate the appropriateness of spatially related data.
Determine if a written agreement is required with the provider of GIS data.
Adhere to issues of confidentiality when spatial data are considered sensitive (e.g., locations of crime incidents and private residences).
Seek legal counsel when obtaining certain types of GIS data through private companies.
Ensure available metadata is created for acquired GIS data so that data quality can be determined.
Obtain accurate and complete road files for geocoding — address locations can vary with different data sources.
Be aware that data sources will vary in completeness, scale, and accuracy and may include spatial or temporal problems.
Differentiate between distances measured “as the crow flies” and distances measured along road networks.
Consider GIS usefulness in comparing what is actually in the environment to what is perceived by the individual to be in the environment.
Develop GIS data that are appropriate to the research question.
Keep in mind that GIS is effective in bringing together disparate data sources to measure a variety of environmental supports for physical activity.
Realize that GIS does not differentiate between good and bad data — therefore, GIS can make attractive but inaccurate products!
