# Erratum, Vol. 1, No. 4

**Published:** 2004-12-15

In the PDF version of the article “Considerations for Using a Geographic Information System to Assess Environmental Supports for Physical Activity,” Figure 2 was incorrectly labeled. The correct figure legend is “Figure 2. Using a half-mile buffer to represent a neighborhood around a survey respondent’s home address, GIS can be used to identify a sidewalk or recreation facility in a survey respondent’s neighborhood.” The correct legend for Figure 2 appeared in the original HTML version, and the PDF version was corrected on our Web site on October 12, 2004. It appears online at http://www.cdc.gov/pcd/issues/2004/oct/04_0047.htm. We regret any confusion this error may have caused.

